# Improving T-DNA Transfer to *Tamarix hispida* by Adding Chemical Compounds During *Agrobacterium tumefaciens* Culture

**DOI:** 10.3389/fpls.2020.501358

**Published:** 2020-09-29

**Authors:** Huimin Zhao, Yaqi Jia, Yanting Cao, Yucheng Wang

**Affiliations:** State Key Laboratory of Tree Genetics and Breeding, Northeast Forestry University, Harbin, China

**Keywords:** *Agrobacterium*-mediated transformation method, spermidine (Spe), azacitidine (5-AzaC), dithiothreitol (DTT), acetosyringone (AS), T-DNA transfer, *Agrobacterium* infection

## Abstract

*Agrobacterium tumefaciens*-mediated gene transfer is the most commonly used method for plant genetic engineering. However, during the period of *A. tumefaciens* culture, the effects of *Agrobacterium* culture before inoculation on genetic transformation are poorly understood. In the present study, we investigated the factors that affect the genetic transformation efficiency during *Agrobacterium* culture using *Tamarix hispida* as transgenic plant material. *Agrobacterium* treatment with spermidine (Spe), azacitidine (5-AzaC), dithiothreitol (DTT), or acetosyringone (AS) alone all significantly improved the efficiency of T-DNA transfer. Treatment with 5-AzaC reduced DNA methylation in *Agrobacterium* to induce the expression of virulence (*vir*) family genes, including *vir*A, *vir*B1, *vir*C1, *vir*D2, *vir*D4 *vir*E2, and *vir*G. Spe treatment significantly induced the expression of all the studied genes, including *vir*A, *vir*B1, *vir*C1, *vir*D1, *vir*D2, *vir*D4, *vir*E2, and *vir*G. DTT treatment decreased reactive oxygen species accumulation. AS treatment activated the expression of the genes *vir*A, *vir*B1, *vir*C1, *vir*D1, *vir*D2, *vir*D4 and *vir*G. All these effects resulted in increased T-DNA transfer. Additionally, combined Spe, 5-AzaC, DTT, and AS treatment improve *Agrobacterium* infection to a greater extent compared with their use alone, increasing T-DNA transfer by more than 8-fold relative to no treatment. Therefore, to improve genetic transformation, pretreatment of *Agrobacterium* during the culture period is important for improving genetic transformation efficiency.

## Introduction

Genetic transformation is a method that transfers DNA of interest into the cell of an organism resulting in genetic alteration, and has been used in many areas of biotechnology, such as studies of gene function, genetic improvement, and molecular breeding. To deliver foreign genes into host plants, three methods are mainly used: *Agrobacterium tumefaciens-* or *Agrobacterium rhizogenes-*mediated plant transformation (Hinchee et al., 1988), protoplast transformation (Fischer and Hain, 1995), and particle bombardment (McCabe et al., 1988). Among these methods, *Agrobacterium*-mediated plant genetic transformation has low cost, is the best choice for plant transformation, and is the predominant method used to generate genetically modified plants. Most transgenic plants (85%) were generated by using *Agrobacterium*-mediated transformation (Yu et al., 2010). This genetic transformation method has the advantages of simple operation, high reproducibility, low copy number of transgene in the host genome, easier manipulation, and transfer of larger fragments of DNA (Kohli et al., 2003; Ishizaki et al., 2008). However, the efficiency of *Agrobacterium*-mediated plant genetic transformation could be improved. Transgene delivery mediated by *Agrobacterium* is affected by many factors, such as explant type, *Agrobacterium* strain, *Agrobacterium* concentrations, *Agrobacterium* suspension medium, the physiological status of the host plant species, and co-cultivation conditions. To improve the efficiency of transgene delivery, multiple factors involved in transformation need to be considered. For instance, phenolic compounds can activate the *vir* genes to regulate T-DNA transport, and several studies have shown that the addition of a phenolic compound such as acetosyringone (AS), into the co-cultivation medium could highly improve transgenic efficiency (Rashid et al., 1996; Wenck et al., 1999; Rohini and Rao, 2000). During the *Agrobacterium* infection period, the infected plants will induce a pathogen defense response to generate reactive oxygen species (ROS), and supplementation with of ROS scavenging factors in the co-cultivation medium, such as dithiothreitol (DTT), sodium thiosulfate (STS), and L-cysteine (L-Cys), can reduce ROS accumulation and increase the efficiency of transgene delivery. For instance, Sivanandhan et al. (2015) added thiol compounds, including DTT at 75 mg/l, STS at 125 mg/l, and L-Cys at 100 mg/l, to the co-cultivation medium, resulting in increased rates of genetic transformation. In addition, to improve T-DNA transfer, vacuum infiltration can be used, which could infiltrate plants in a short period of time and is more robust (Chen et al., 2014).

Su et al. (2018) added Spe to the culture medium during *Vitis vinifera* somatic embryo growth, which increased transgenic resistant somatic embryos induction rate, suggesting that Spe plays a positive role in T-DNA transfer. The above studies significantly improved the efficiency of T-DNA transfer and indicated that the importance of activating the T-DNA transfer capability of *Agrobacterium.*

However, most previous studies on improvement of T-DNA transfer assessed the period of the transformation and co-cultivation. The treatments were mainly conducted during co-cultivation time or were added to the co-cultivation medium. In the present study, we identified and studied factors that might affect the infection capability of *Agrobacterium*. Our results also indicated that treatment of *Agrobacterium* in culture before inoculation is relatively important for T-DNA transfer efficiency and should be considered in *Agrobacterium-*mediated transformation studies.

## Materials and Methods

### Plant Material and Grown Conditions

The seeds of *T. hispida* were collected from the Turpan Botanical Garden of the Chinese Academy of Sciences. The seeds of *T. hispida* were surface-sterilized in 33% (v/v) sodium hypochlorite and grown on Murashige-Skoog (MS) solid medium containing 2% sucrose for germination. Two-month-old seedlings were used for transient transformation investigation.

### Treatments of *Agrobacterium tumefaciens* During Culture

A single colony of *Agrobacterium tumefaciens* (EHA105) harboring pCAMBIA1301 was picked and cultured in LB liquid culture supplied with 100 μg/ml rifampicin and 50 μg/ml Kanamycin at 28°C until the OD_600_ reached 0.8 (about 24 h). The culture was diluted 100-fold to a volume of 50 ml and used for the following treatments. To determine the effects of Spe, the diluted cultures were supplied with 0, 1, 3, 5, and 7 mM Spe, and then cultured at 28°C until the OD_600_ reached 0.6–0.7 (about 7–8 h). For 5-AzaC treatment, the diluted cultures were added with 0, 10, 20, and 30 μM5-AzaC, and cultured at 28°C until the OD_600_ reached 0.6–0.7 (about 7–8 h). To test the effect of AS, the diluted cultures containing 0, 100, 120, 150, and 200 μM AS were cultured at 28°C until the OD_600_ reached 0.6–0.7 (about 7–8 h). For DTT supplementation, the diluted cultures were supplied with 0, 50, 100, and 150 μM DTT, and cultured at 28°C until the OD_600_ reached 0.6–0.7 (about 7–8 h). After cultivation, the cultures were centrifuged at 2,800 × g to pellet the *Agrobacterium* cells, and then used for transient transformation.

### *Agrobacterium* Mediated Transient Transformation

Transient transformation mediated by *Agrobacterium* was performed according to the method described by Zang et al. (2017). Briefly, the pellet of *A. tumefaciens* EHA105 cells were centrifuged at 2,800 × *g* for 10 min, and then adjusted to an OD_600_ of 0.8 using a solution comprising 1/2 MS +10 mM CaCl_2_+.5% (w/v) sucrose + 100 μM acetosyringone + Tween20 (0.02%, v/v), pH 5.8 (transformation solution). The plants were soaked in the transformation solution with shaking at 90 rpm for 2 h at 25°C. The plants were then washed quickly with distilled water for 10 s to remove the excessive *A. tumefaciens* cells. After washing, the plants were planted vertically on 1/2 MS solid medium [1/2 MS+1% (w/v) sucrose+100 μM acetosyringone, pH 5.8]. After culture for 72 h, the plants were washed with distilled water and wiped with sterile filter paper to remove excessive water. These plants were harvested for GUS staining and GUS activity assays. Each sample contains at least 10 seedlings, and three biological repeats were performed.

### Determination of β-Glucuronidase (GUS) Activity

GUS activity was measured following the method described by Jefferson et al. (1987). Briefly, the samples were ground into a fine powder under liquid nitrogen, and then homogenized in an extraction buffer (50 mM NaH_2_PO_4_-Na_2_HPO_4_, pH 7.4, 10 mM β-mercaptoethanol, 10 mM EDTA, 0.1% Triton X-100, 0.1% sodium lauryl sarcosine). Then, 1 mM 4-methylumbelliferyl-β-d-glucuronide (MUG) was added into the extraction buffer, the enzyme reaction was performed at 37°C, and was stopped by adding 500 μl of 0.2 M Na_2_CO_3_. After stopping the enzyme reaction, the fluorescence of 4-methylumbelliferone was checked using a DyNA Quant fluorometer (Hoefer Pharmacia Biotech Inc., San Francisco, CA), and a protein standard curve was generated using the Bradford assay to calculate the GUS activity.

### Determination of the Soluble ROS Content and DNA Methylation Level

The ROS content was determined using a Hydrogen Peroxide Content measuring kit (Nanjing Jiancheng Bioengineering Institute, Nanjing, China), according to the manufacturer’s protocol. The DNA methylation content was determined using Methylated DNA Quantification Kit (Fluorometric) according to the manufacturer’s protocol (Epigentek Group Inc., China). Three independent biological replications were performed.

### Quantitative Real-Time Reverse Transcription PCR (qRT-PCR)

Total RNA from *T. hispida* or *Agrobacterium* cells was isolated using the Trizol reagent (Promega) and treated with DNaseI (RNase free) to remove DNA contamination. The integrality of RNA was analyzed using agarose gel electrophoresis. For the synthesis of *T. hispida* cDNA, 1 μg of total RNA from each sample was reverse-transcribed using a random primer (9 mer) as synthesis primers with a PrimeScript™ RT reagent Kit (Takara, China) in volume of 10 μl according to its instruction. The products were subsequently diluted to 100 μl and used as the template for qRT-PCR. For *T. hispada*, two internal references were used, including β-tubulin (GenBank number: FJ618519) and α-tubulin (GenBank number: FJ618518). For *Agrobacterium* cells, 16S rRNA was used as internal reference. All the primers used are shown as **Table S1**. qRT-PCR was performed on a qTOWER2.0 (Analytic Jena, Germany). The qRT-PCR reaction mixture contained 10 μl of SYBR Green Real-time PCR Master Mix (Takara), forward and reverse primers (0.5 μM each), and cDNA template (2 μl cDNA that is equivalent to the transcript from total RNA 20 ng) in a volume of 20 μl. qRT-PCR was conducted according to the following cycling parameters: 94°C for 30 s; followed by 40 cycles at 94°C for 10 s, 59°C for 30 s, and 72°C for 40 s. Three independent biological replicates were performed, and the expression levels were calculated from the Ct (cycle threshold) using the 2^-ΔΔ^Ct method (Livak and Schmittgen, 2001)

### Statistical Analysis

Statistical analyses were carried out using the SPSS 16.0 software package (SPSSInc, Chicago, IL, USA). data were compared using one-way analysis of variance (ANOVA) and differences were considered statistically significant at P < 0.05.

## Results

### The Effects of Azacitidine (5-AzaC) on T-DNA Transfer, DNA Methylation, and the Expression of Virulence (*vir*) Genes

**Figure 1:**
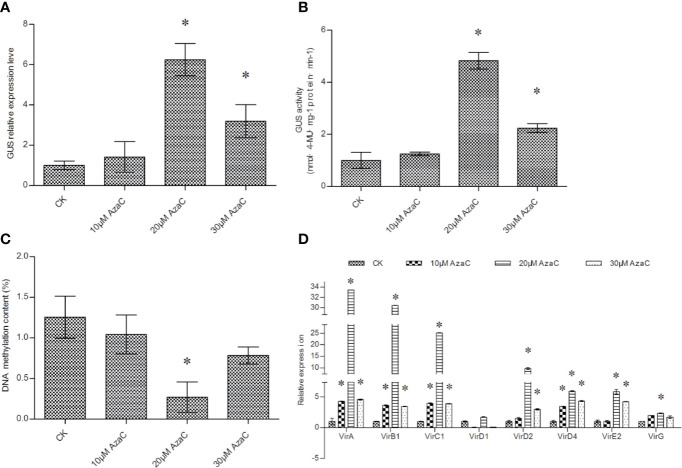
The efficiency of T-DNA transfer induced by 5-AzaC treatment. **(A)** Analysis of *GUS* expression to determine the effects of 5-AzaC treatment of *Agrobacterium tumefaciens* cells. **(B)** GUS activity analysis of *T. hispida* plants transiently transformed using *Agrobacterium* cells treated with 5-AzaC. **(C)** The effects of 5-AzaC on total DNA methylation in *Agrobacterium* cells. DNA methylation was analyzed on *Agrobacterium* cells treated with different concentrations of 5-AzaC. **(D)** The relative expression of *vir* family genes in *Agrobacterium* cells in response to 5-AzaC supplements. The relative expression was calculated as the transcript level at different treatments divided by the transcript level without treatment (0 μM). LB medium was added with 0, 10, 20, or 30 mM 5-AzaC to culture *Agrobacterium* cells before genetic transformation, and qRT-PCR was performed to determine the expression of the *vir* genes. Data are means ± SD from three independent experiments. (*) indicates a significant difference with the wild-type (WT) at P < 0.05.

*Agrobacterium* cells treated with 5-AzaC before transformation significantly elevated β-glucuronidase (GUS) expression and activity in transient transformed *T. hispida* plants (**Figures 1A, B**). GUS expression and activity was not significantly activated by 10 μM 5-AzaC treatment but was by 20 and 30 μM AzaC treatment. The expression and activity of GUS reached peak levels after treatment with 20 μM 5-AzaC (**Figures 1A, B**). 5-AzaC is a DNA demethylation agent; therefore, treatment with 5-AzaC may decrease DNA methylation in *Agrobacterium* cells. The level of DNA methylation was investigated in *Agrobacterium* cells after 5-AzaC treatment. The results showed that 20 and 30 μM 5-AzaC treatment significantly decreased DNA methylation; however, 20 μM 5-AzaC reduced DNA methylation to a greater extent than 30 μM AzaC (**Figure 1C**), which is consistent with the trend of T-DNA transfer efficiency (**Figure 1A**).

DNA methylation usually correlates with gene expression; therefore, we further studied the expression of different *vir* genes in response to 5-AzaC treatment. The results showed that 10, 20, and 30 μM 5-AzaC all significantly induced the expression *vir* genes. However, 20 μM 5-AzaC treatment activated the expression of *vir* genes more potently than 10 and 30 μM 5-AzaC The *vir*A, *vir*B1, *vir*E2, *vir*C1, *vir*D2, *vir*D4, and *vir*G genes were all significantly activated by 20 μM 5-AzaC treatment (**Figure 1D**).

### The Effects of Spermidine (Spe) on T-DNA Transfer and the Expression of *vir* Family Genes

**Figure 2:**
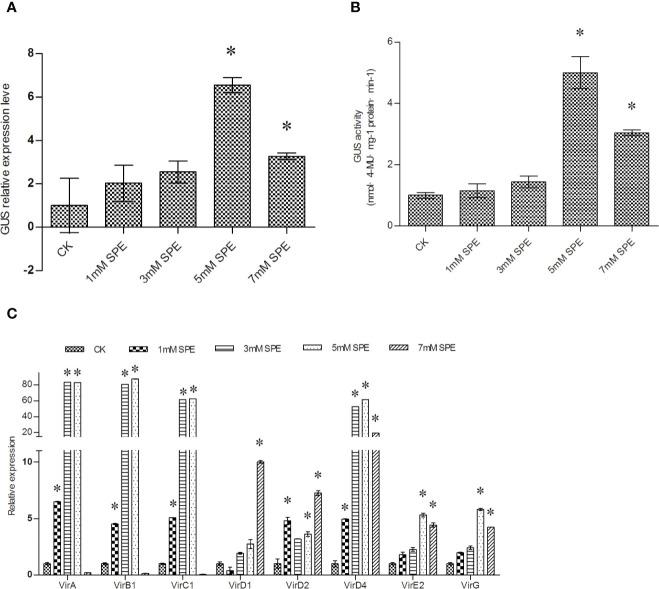
T-DNA transfer efficiency was improved when *Agrobacterium* cells were treated with spermidine (Spe). **(A)** The effect of Spe treatment on the efficiency of T-DNA transfer was determined *via GUS* expression analysis; **(B)** GUS activity analysis on the transformed *T. hispida* plants using *Agrobacterium* cells treated with Spe. **(C)** The relative expression of *vir* genes in *Agrobacterium* cells when treated with different concentrations of Spe. The relative expression was calculated as the transcript level at different treatments divided by the transcript level without treatment (0 mM Spe). For *A. tumefaciens* treatment, 0, 1, 3, 5, or 7 mM Spe was added into LB medium before genetic transformation, and qRT-PCR was performed to determine the expression of the *vir* genes. Data are means ± SD from three independent experiments. (*) indicates a significant difference with the wild-type (WT) at P < 0.05.

To study the effect Spe treatment on T-DNA transfer, different concentrations of Spe were supplied in Luria-Bertani (LB) medium for *Agrobacterium* cultivation. The cultures were harvested for transient transformation. After transient transformation, T-DNA transfer efficiency was analyzed by determining GUS expression in the transiently transformed plants. Compared with the control (transient transformation with untreated *Agrobacterium* cells), supplementation with 5 and 7 mM Spe significantly increased GUS expression and activity (**Figures 2A, B**). GUS expression and activity increased to a greater extent under 5 mM Spe than under 7 mM Spe (**Figures 2A, B**). These results suggested that supplementation with Spe at the appropriate level can significantly improve genetic transformation.

To further study the mechanism of the improved genetic transformation mediated by Spe treatment, the expression levels of different *vir* genes were studied. The results showed that except for *vir*D1, all the studied *vir* genes were significantly induced by treatment with 5 mM Spe (**Figure 2C**). These results indicated that Spe treatment could induce the expression of certain *vir* family genes, leading to improved T-DNA transfer efficiency.

### The Effects of DTT on T-DNA Transfer and ROS Accumulation

**Figure 3:**
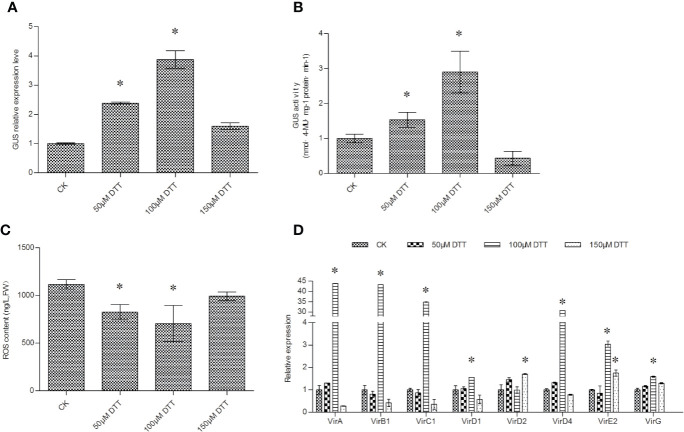
DTT treatment of *Agrobacterium* cells improves the efficiency of T-DNA transfer. **(A)** The effect of DTT treatment on the efficiency of T-DNA transfer was determined *via GUS* expression analysis; **(B)** GUS activity analysis on the transiently transformed *T. hispida* plants. **(C)** ROS accumulation in *Agrobacterium* cells in response to treatment with different concentrations of DTT. **(D)** The relative expression of *vir* genes in *Agrobacterium* cells when treated with different concentrations of DTT. The relative expression was calculated as the transcript level at different treatments divided by the transcript level without treatment (0 μM DTT). For treatment, 0, 50, 100, or 150 μM of DTT was added into LB medium to culture *A. tumefaciens* before genetic transformation, and qRT-PCR was performed to determine the expression of the *GUS* gene. Data are means ± SD from three independent experiments. (*) indicates a significant difference with the wild-type (WT) at P < 0.05.

Different concentrations of DTT were used in the culture of *Agrobacterium* before transient transformation. GUS expression and activity measurements showed that supplementation with DTT could significantly improve T-DNA transfer. DTT at concentrations from 50 to 150 μM could significantly increase T-DNA transfer. The transformation efficiency reached a peak at 100 μM DTT (**Figures 3A, B**).

DTT plays a role in ROS scavenging; therefore, the ROS concentration in *Agrobacterium* cells was determined. The results showed that 50, 100, and 150 μM DTT all significant reduced ROS accumulation in *Agrobacterium* cells (**Figure 3C**). The decreased ROS accumulation would reduce the damage to *Agrobacterium* cells, which might contribute to increased T-DNA transfer. In addition, supplement with DTT during culture period can induce the expression of all studied *vir* genes, especially 100 μM DTT was added (**Figure 3D**), which is consistent with the results that 100 μM DTT could highly activate T-DNA transformation.

### Acetosyringone (AS) Treatment Could Significantly Induce *vir* Gene Expression to Increase T-DNA Transfer

**Figure 4:**
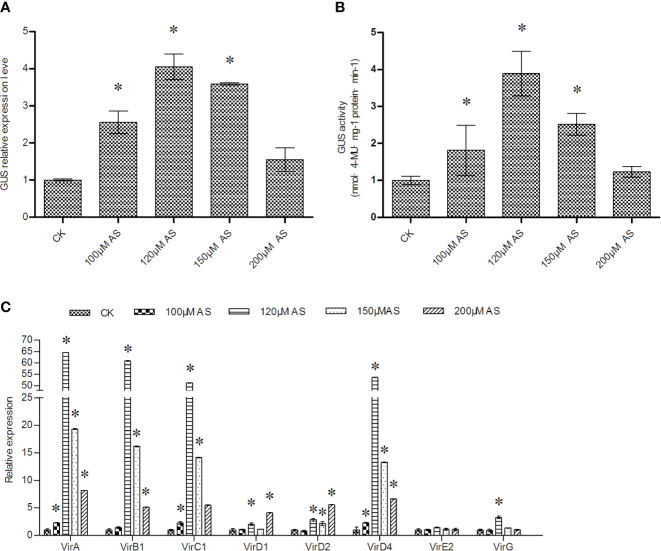
The effects of AS treatment on T-DNA transfer efficiency. **(A)** Analysis of *GUS* expression to determine the effects of AS treatment of *Agrobacterium* cells; **(B)** GUS activity analysis in *T. hispida* plants transiently transformed with *Agrobacterium* treated with AS. **(C)** The relative expression of *vir* family genes in *Agrobacterium* cells treated with different concentrations of AS. The relative expression was calculated as the transcript level at different treatments divided by the transcript level without treatment (0 μM AS). To culture *A. tumefaciens*, 0, 100, 120, 150, or 200 μM AS were added separately into LB medium before genetic transformation, and qRT-PCR was performed to determine the expression of the *vir* genes. Data are means ± SD from three independent experiments. (*) indicates a significant difference with the wild-type (WT) at P < 0.05.

AS was added into LB medium to culture *Agrobacterium* cells before transient transformation. Supplementation with AS from 100 to 200 μM could significantly improve T-DNA transfer, with 120 μM AS treatment producing the largest effect (**Figures 4A, B**). The expression of *vir* genes induced by AS were further studied. The results showed that compared with other AS concentrations, 120 μM AS treatment could significantly induce the expression of all *vir* genes, except *vir*E2. In particular, 120 μM AS treatment induced *vir* gene expression to a greater extent than 100 and 150 μM AS. However, 200 μM AS treatment only induced high expression of *vir*D1 and *vir*D2 compared with 150 μM AS treatment (**Figure 4C**). These results indicated that AS treatment during *Agrobacterium* cell culture could induce the expression of *vir* genes to improve T-DNA transfer.

### The Combined Effects of SPE, AS, DTT, and 5-AzaC on T-DNA Transfer

**Figure 5:**
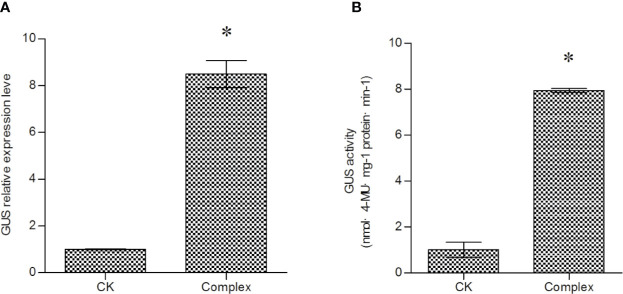
The combined effect of 5-AzaC, DTT, AS, and Spe on T-DNA transfer. **(A)** Investigation of T-DNA transfer efficiency induced by combined treatment with 5-AzaC, DTT, AS, and Spe; the expression of the *GUS* gene was analyzed to determine T-DNA transfer efficiency. **(B)** Analysis of GUS activity in transiently transformed *T. hispida* plants. For *A. tumefaciens* treatment, 20 mM 5-AzaC, 120 μM AS, 5 mM Spe, and 100 μM DTT were added together into LB medium to culture *A. tumefaciens* before genetic transformation, and qRT-PCR was performed to determine the expression of the *GUS* gene. Data are means ± SD from three independent experiments. (*) indicates a significant difference with the wild-type (WT) at P < 0.05.

The effects of a combination of Spe, 5-AzaC, DTT and AS on T-DNA transfer were investigated. The mixture of 100 μM DTT, 20 μM 5-AzaC, 5 mM SPE, and 120 μM AS were added into LB medium to culture *Agrobacterium* cells before transformation. Both GUS expression and GUS activity measurements showed that the combined treatment could increase the efficiency of T-DNA transfer to a greater extent than any of the reagents used alone, and could improve *GUS* expression by more than eight-fold (**Figures 5A, B**). This result indicated that DTT, Azac, SPE, and AS could work coordinately to activate T-DNA transfer.

Our results showed that treatment of *Agrobacterium* cells before genetic transformation could significantly increase the T-DNA transfer efficiency, and 5-AzaC, Spe, DTT, and AS all exert positive roles to improve T-DNA transfer. Combined use of 5-AzaC, Spe, DTT, and AS improved T-DNA transfer by more than eight-fold, suggesting that treatment of *Agrobacterium* cells before genetic transformation is important and should be used in genetic transformation.

## Discussion

Transient transformation can accurately reflect the efficiency of T-DNA transfer. In addition, because transfer of T-DNA into cells is the first step to obtain stable transformation, determining of the efficiency of transient transformation could reflect the efficiency of stable genetic transformation. Wild type of *A. tumefaciens* is a gram-negative bacterium that can transfer genes into host plants, which leads to tumors. The Ti plasmid in *A. tumefaciens* contains different *vir* family genes and a transfer DNA (T-DNA) region. *Vir*A and *vir*G families serve as transcriptional activators for *vir* gene transfer and transformation of higher plants (Gao and Lynn, 2005). The *vir*B proteins and the *vir*D4 protein can form a membrane complex to mediate the transfer of T-DNA to plant cells (Vergunst et al., 2005). *Vir*G serve as a positive regulatory protein that can activate *vir* gene expression together with plant molecules (Chen et al., 1991). Michielse et al. (2005) showed that during gene transformation, virC and virD excise the T-DNA region from the Ti plasmid, and then the excised T-DNA forms a T-DNA complex with *vir*D and *vir*E2 to enter host plant cells. The T-DNA finally enters the plant nucleus, and the *vir*D and *vir*E2 proteins are stripped from T-DNA complex when the T-DNA enters the cell nucleus (Nonaka et al., 2017). In the present study, the results showed that during the period of *Agrobacterium* cell culture, treatment with 5-AzaC, Spe, and AS all highly induce *vir* gene expression (**Figures 1** **–** **4**), suggesting that these factors might improve T-DNA transfer by directly activating the *vir* genes.

Azacytidine is a DNA demethylating agent that can reduce or inhibit DNA methylation. Palmgren et al. (1993) showed that *Agrobacterium* cells treated with 5-AzaC showed significantly increased transient expression efficiency, and AzaC also inhibits the methylation-dependent inactivation of the transformed gene. These results suggested that 5-AzaC treatment could improve the genetic transformation efficiency. The results of the present study showed that 5-AzaC treatment could reduce the methylation of *Agrobacterium* DNA (**Figure 1**), which leads to the increased expression of *vir* genes, which ultimately improved the transformation efficiency. Therefore, reduction of methylation of *Agrobacterium* DNA is important for enhancing T-DNA transfer.

Kumar and Rajam (2005) showed that treatment of *Agrobacterium* cells with polyamine prior to transformation, including putrescine and Spe, could induce T-DNA transfer. These results showed that Spe plays a role in inducing genetic transformation. In the present study, Spe treatment of *Agrobacterium* cells highly induced T-DNA transfer in *T. hispida* (**Figure 2A**); at the same time, all the studied *vir* genes were significantly induced by Spe (**Figure 2C**). These results suggested that Spe could improve T-DNA transfer by inducing the expression of *vir*A, *vir*B1, *vir*C1, *vir*D1, *vir*D2, *vir*D4, *vir*E2, and *vir*G gene.

DTT is an antioxidant that efficiently prevents oxidation of nucleic acids by phenolic compounds and has been used to stabilize enzymes and other proteins containing sulfhydryl groups (Thanh et al., 2009; Chen et al., 2011). Supplement with DTT during inoculation period could improve genetic transformation (Ahn et al., 2013; Li et al., 2017). Our results showed that DTT treatment could significantly reduce ROS accumulation in *Agrobacterium* cells, and the ROS level was negatively correlated with the transformation efficiency (**Figure 3C**). These results suggested that ROS accumulation might damage *Agrobacterium* cells to decrease their infection capability. Therefore, reducing ROS accumulation by DTT treatment would improve T-DNA transfer efficiency in *T. hispida*. Therefore, DTT is also an important genetic transformation inducer.

Our results showed that treatment with 5-AzaC or Spe increased T-DNA transfer compared with treatments DTT and AS (**Figures 1** **–** **4**). However, AS is widely used to induce efficient transformation in *A. tumefaciens*-mediated genetic transformation. These results indicated that 5-AzaC and Spe might be superior to AS in the induction of T-DNA transfer during *Agrobacterium* cell culture. 5- AzaC at 20 μM or 5 mM Spe are the most suitable concentrations to improve T-DNA transfer (**Figures 1A**, **2A**), and 120 μM AS is also the most suitable concentration for improving T-DNA transfer (**Figure 4A**). However, 20 μM AzaC and 5 mM Spe both improved T-DNA transfer to a greater extent than 120 μM AS (**Figures 1A**, **4A**). In addition, treatment with 5-AzaC seems to be more efficient in inducing T-DNA transfer, because 20 μM AzaC treatment could induce T-DNA transformation more efficiently than 120 μM AS in *T. hispida* (**Figures 1D**, **4C**). Therefore, 5-AzaC should be considered for use in *A. tumefaciens*-mediated transformation rather than AS.

## Data Availability Statement

The datasets generated for this study are available on request to the corresponding author.

## Author Contributions

YW conceived and directed the project. HZ performed the experiments. YC and YJ performed the overall data analysis. HZ and YW wrote the manuscript. All authors contributed to the article and approved the submitted version.

## Funding

This work was financially supported by the National Natural Science Foundation of China (No. 31770704). Heilongjiang Touyan Innovation Team Program (Tree Genetics and Breeding Innovation Team) and the Overseas Expertise Introduction Project for Discipline Innovation (B16010).

## Conflict of Interest

The authors declare that the research was conducted in the absence of any commercial or financial relationships that could be construed as a potential conflict of interest.
