# Supplementary material for: Improving T-DNA Transfer to Tamarix hispida by Adding Chemical Compounds During Agrobacterium tumefaciens Culture
Source: Front Plant Sci. 2020 Sep 29;11:501358. doi: 10.3389/fpls.2020.501358 (PMC7550641; doi:10.3389/fpls.2020.501358)
Supplement: Supplementary file 1 [file Table_1.docx]

**Table S1.** The primers used for qPCR

| Genes | GenBank number | Forward Primers (5’-3’) | Reverse Primers (5’-3’) |
| --- | --- | --- | --- |
| virA | [AAZ50512.1](https://www.ncbi.nlm.nih.gov/protein/71849564) | GCGCATGGTGTTATGCCACC | GCCCAACAGTTGAACTAACG |
| virB1 | [AAZ50518.1](https://www.ncbi.nlm.nih.gov/protein/71849570) | GCGATTCATGACAACACGAC | CAGCATGTTTGCAGCGGCAG |
| virD4 | [AAZ50535.1](https://www.ncbi.nlm.nih.gov/protein/71849587) | GCGTACAAGTATTTATGGCC | GTCGCCCTTTGATGAGAACG |
| virD2 | AAZ50533.1 | TGAATATCGACGCCTTGAGC | GCTCGCTCTCCAATGGTACG |
| virC1 | AAZ50531.1 | GCTAACGCCGCTCGACATCG | GCATCTCTTTCATGCATGGG |
| virD1  virE2  virG  16S RNA  a-tubulin  β-tubulin  GUS | AAZ50532.1  [AAZ50538.1](https://www.ncbi.nlm.nih.gov/protein/71849590) [AAZ50529.1](https://www.ncbi.nlm.nih.gov/protein/71849581)  M11223.1  FJ618518  FJ618519   LC122496.1 | GGGTTCAAGGTCGTGAGTGC  GGAAAAATGGGGGATGATGG TGAGATCGTTCGAAATCTGG  ATTCGGAGGAACACCAGTGG CACCCACCGTTGTTCCAG  GGAAGCCATAGAAAGACC  GTCGCGCAAGACTGTAACCA | TAGAAGCATGGATACATTGC  ATATCGGGCAGCAGCGAACC  CGGACATCAAGCGACGTTGC CCTTTGAGTTTTAATCTTGC ACCGTCGTCATCTTCACC  CAACAAATGTGGGATGCT TGGTTAATCAGGAACTGTTG |
